# Mitochondrial complex I *NUBPL* mutations cause combined dystonia with bilateral striatal necrosis and cerebellar atrophy

**DOI:** 10.1111/ene.13956

**Published:** 2019-04-20

**Authors:** B. Balint, G. Charlesworth, M. Stamelou, L. Carr, N. E. Mencacci, N. W. Wood, K. P. Bhatia

**Affiliations:** ^1^ Department of Clinical and Movement Neurosciences UCL Queen Square Institute of Neurology London UK; ^2^ Department of Neurology University Hospital Heidelberg Heidelberg Germany; ^3^ Department of Neurology Charing Cross Hospital London UK; ^4^ Second Department of Neurology Attiko Hospital University of Athens Athens Greece; ^5^ Parkinson's Disease and Movement Disorders Department HYGEIA Hospital Athens Greece; ^6^ Neuroscience Department GOSH London UK; ^7^ Department of Neurology Northwestern University Chicago IL USA; ^8^ Department of Molecular Neuroscience Institute of Neurology University College London London UK

**Keywords:** ataxia, autosomal recessive, bilateral striatal necrosis, dystonia, NUBPL

## Abstract

**Background and purpose:**

The recent advances in genetics have helped to unravel the cause of many dystonia syndromes. With the broadening spectrum of genetically defined dystonia syndromes, distinct clinico‐radiological phenotypes are a welcome handle to guide the diagnostic work‐up.

**Methods:**

Exome sequencing was used to elucidate the genetic cause of a syndrome characterized by generalized dystonia, pyramidal and cerebellar involvement, with bilateral striatal necrosis (BSN) and cerebellar atrophy on magnetic resonance imaging. Homozygosity mapping and linkage analysis were used in a supportive role. Known genetic causes of BSN were excluded by use of exome data or Sanger sequencing.

**Results:**

Compound heterozygous mutations were identified in the *NUBPL* gene in a small UK kindred. The gene lay in a region of positive linkage and segregated with disease in a family of six individuals.

**Conclusion:**

*NUBPL* mutations cause early onset, autosomal recessive generalized dystonia with cerebellar ataxia, pyramidal signs, preserved cognition and a distinct magnetic resonance imaging appearance with BSN and cerebellar atrophy.

## Introduction

With the advances of genetic techniques, an increasing number of genetically defined dystonia syndromes are recognized 1. Using whole exome sequencing, linkage analysis and homozygosity mapping, *NUBPL* mutations are now identified as the cause of autosomal recessive dystonia combined with cerebellar ataxia and pyramidal involvement in a UK kindred.

## Methods

### Standard protocol approvals, patient consents and genetic analysis

Affected and non‐affected individuals of a Caucasian UK kindred were examined by neurologists specialized in movement disorders at the National Hospital of Neurology, Queen Square, London. The study was approved by the University College London Research Ethics Committee and was conducted in agreement with the Declaration of Helsinki. Study participants gave written informed parental and patient consent. Details of genetic analysis, including genome‐wide genotyping by DNA array chip, homozygosity mapping, linkage analysis and variant filtration can be found in Appendix S1.

## Results

### Clinical description

**Figure 1:**
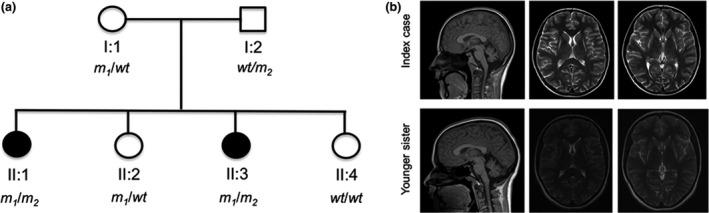
(a) Genetic pedigree of the family with mutational status. All individuals were examined clinically and provided samples for DNA extraction. There was no report of similar disease in either parent's extended families. Mutational status for the p.L104P (m_1_) and p.D96V (m_2_) mutations in *NUBPL* are shown below each individual, with wt indicating a wildtype allele. (b) Brain MRI showing bilateral striatal necrosis and cerebellar atrophy in the index case and her sister. Sagittal fluid‐attenuated inversion recovery (left) showing cerebellar atrophy in the index case and the sister. Transversal T2‐weighted images (middle and right) showing bilateral putaminal signal abnormality and atrophy, and symmetrical loss of posterolateral putaminal volume and replacement by gliotic looking tissue.

The index patient is a 25‐year‐old woman, the older of two affected sisters. Two other sisters and the non‐consanguineous parents are unaffected (pedigree in Fig. 1a). She was hypotonic at birth and had delayed motor milestones (sitting, 2 years; walking, 7 years). Subsequently, her walking deteriorated and she lost independent walking by age 11. In her teenage years, her speech and her upper limb function deteriorated. On examination (Video S1), she was wheelchair‐bound with generalized dystonia, involving inability to stand or walk alone, marked impairment of hand use, and dystonic grimacing of the face. Her speech was markedly dysarthric. There was gaze evoked nystagmus bilaterally. Pyramidal signs comprised hyperreflexia with crossed adductor response and distal grade 3–4 weakness. Her sister (17 years) was similarly although less severely affected by dystonia. She had more prominent gait ataxia and an intention tremor. Brain magnetic resonance imaging (MRI) of both showed bilateral putaminal atrophy with T2 hyperintensities and cerebellar atrophy (Fig. 1b).

### Genetic analysis

After whole exome sequencing and filtration (Appendix S1), only one potentially causative compound heterozygous mutation remained: the affected gene *NUBPL* is involved in complex I assembly within the mitochondria. Using the longest transcript (ENST00000281081) as a reference, the first variant (c.311T>C; p.L104P) affects a conserved base [GERP = 5.4 (max = 6)] and is predicted to be highly damaging by all four *in silico* prediction programs used (SIFT = 0; Provean = −6.5; PolyPhen2 = 1; MutationTaster = 0.99). It is located adjacent to a previously reported pathogenic mutation in this gene (p.D105Y) 2. The variant is annotated in the gnomAD browser (http://gnomad-old.broadinstitute.org/; last accessed 13 November 2018) with a minor allele frequency of 0.0001461, having been detected 41 times in the heterozygous state in the 280 624 chromosomes sequenced. It has never been detected in the homozygous state. The second variant (c.287A>T; p.D96V) also affects a conserved base (GERP = 5.41) and is not found in any database of human variation. *In silico* predictions of pathogenicity were contradictory: tolerated by SIFT (0.1) and Provean (−1.96), ‘possibly damaging’ by PolyPhen2 (0.71) and damaging by MutationTaster (0.99). Sanger sequencing demonstrated that the observed mutations segregated with disease in the compound heterozygous state (Fig. 1a).

## Discussion

Autosomal recessive *NUBPL* mutations were identified as the cause of early onset, generalized dystonia with cerebellar ataxia, pyramidal signs, preserved cognition and a distinctive MRI appearance with bilateral striatal necrosis (BSN) and cerebellar atrophy. *NUBPL* blends in with a number of other genes important for mitochondrial function and associated with BSN, as it encodes a protein required for the correct assembly of the respiratory chain complex I. Indeed, mutations in *NUBPL* have previously been associated with disease resulting from complex I deficiency 2, 3, 4. However, the so far associated phenotype, based on the hitherto described eight patients with biallelic *NUBPL* mutations, was more severe, encompassing ataxia, dysarthria and spasticity, variable degrees of cognitive impairment or myopathy, with onset in the first 2 years of life 2, 4. The MRI was considered ‘pathognomonic for the disease’ and comprised abnormalities of the cerebellar cortex, deep cerebral white matter and corpus callosum 2.

Although there is clear clinical (pyramidal, cerebellar dysfunction and dysarthria) and radiological (cerebellar atrophy) overlap between previously reported cases and our patients, there are also significant differences – most notably, predominant generalized dystonia, the presence of BSN on MRI, and the lack of any white matter signal abnormalities in our patients.

The more severe affected status of the previously reported individuals with *NUBPL* mutations may relate to the fact that they carry the same putatively causative genetic mutation on one allele, which is a branch‐point splicing mutation in intron 9 (c.815‐27T>C). This mutation results in a profound decrease in the steady‐state level of *NUBPL* mRNA to 15% of control levels 3. In about half of the cases, the branch site mutation was inherited with a presumed null allele and would therefore be expected to have almost no functional *NUBPL* activity.

The different radiological appearance is explained by previous cases being selected based on an MRI pattern of ‘unclassified leukoencephalopathy’ and absence of signal abnormalities in the basal ganglia, thalami and cerebral cortex.

In summary, the here reported cases expand the clinical and radiological spectrum of *NUBPL* mutations. BSN and cerebellar atrophy might be a useful handle in the differential diagnosis of the long list of early onset combined dystonias.

## Disclosure of conflicts of interest

Bettina Balint is supported by the Robert Bosch Foundation. Gavin Charlesworth received a travel grant by Boston Scientific. Maria Stamelou serves as assistant editor in *Movement Disorders Journal*, has received research support from the Michael J Fox Foundation and travel and speaker honoraria from the Movement Disorders Society, Abbvie Pharmaceuticals, and royalties from Oxford University Press. Lucinda Carr reports no disclosures. Nicholas W. Wood holds grants from the Bachmann–Strauss Dystonia and Parkinson Foundation, the MRC and the Wellcome Trust. Niccolo E. Mencacci reports no disclosures. Kailash P. Bhatia has received grant support from Welcome/MRC, NIHR, Parkinsons UK and EU Horizon 2020. He receives royalties from publication of the *Oxford Specialist Handbook Parkinson's Disease and Other Movement Disorders* (Oxford University Press, 2008), of *Marsden's Book of Movement Disorders* (Oxford University Press, 2012) and of *Case Studies in Movement Disorders – Common and Uncommon Presentations* (Cambridge University Press, 2017). He has received honoraria/personal compensation for participating as consultant/scientific board member from Ipsen, Allergan, Merz and honoraria for speaking at meetings and from Allergan, Ipsen, Merz, Sun Pharma, Teva, UCB Pharmaceuticals and from the American Academy of Neurology and the International Parkinson's Disease and Movement Disorders Society.

## Supporting information


**Figure S1.** Schematic representation of the major steps of the filtration process.

**Video S1.** Video_segment_1: The index case demonstrates generalized dystonia, including the craniocervical region, which worsens on action. Her gait is broad‐based and unsteady. Video_segment_2: The younger sibling has the same phenotype with generalized dystonia and gait imbalance but is less severely affected.

**Appendix S1.** Supplementary methods and results.
**Table S1.** Results of homozygosity mapping.
**Table S2.** Results of linkage analysis.
**Table S3.** Mutational analysis of common genetic causes of BSN.
