# Supplementary figures and images for: Mitochondrial complex I NUBPL mutations cause combined dystonia with bilateral striatal necrosis and cerebellar atrophy
Source: Eur J Neurol. 2019 Apr 20;26(9):1240–3. doi: 10.1111/ene.13956 (PMC6767441; doi:10.1111/ene.13956)

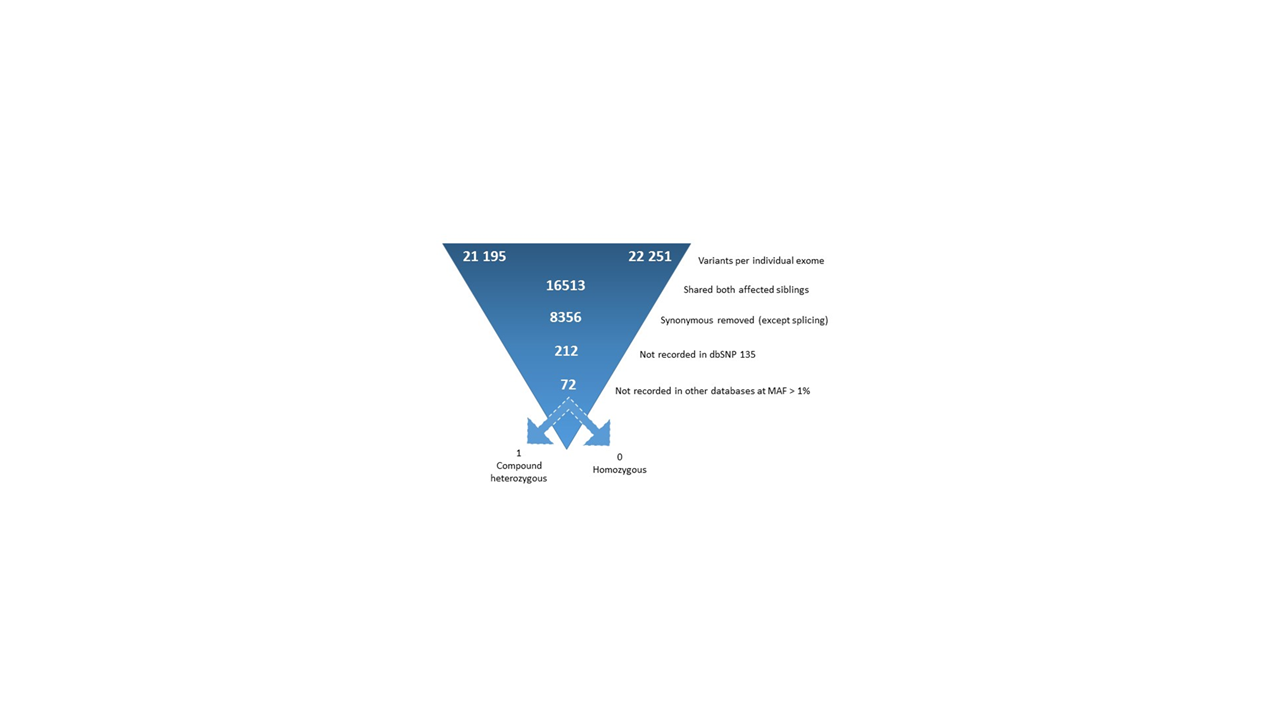

Supplement: Supplementary file 1 — Figure S1. Schematic representation of the major steps of the filtration process. [file ENE-26-1240-s001.tiff]
