# Supplementary material for: Mitochondrial complex I NUBPL mutations cause combined dystonia with bilateral striatal necrosis and cerebellar atrophy
Source: Eur J Neurol. 2019 Apr 20;26(9):1240–3. doi: 10.1111/ene.13956 (PMC6767441; doi:10.1111/ene.13956)
Supplement: Supplementary file 3 — Appendix S1. Supplementary methods and results. Table S1. Results of homozygosity mapping. Table S2. Results of linkage analysis. Table S3. Mutational analysis of common genetic causes of BSN. [file ENE-26-1240-s003.docx]

Supplementary material

**Appendix S1**

**Supplementary methods:**

*Genetic analysis*

DNA from both affected siblings and their mother was used to perform WES, using Illumina’s TruSeq (62Mb) DNA Sample Prep Kit and Exome Enrichment Kit. Subsequently, variants were filtered to identify potentially causal compound heterozygous or homozygous candidates. Genome-wide SNP data was generated using the OmniExpress DNA array and used to perform linkage analysis and homozygosity mapping

*Genome-Wide Genotyping by DNA Array Chip*

*DN*A was obtained from all members of the core index family (Fig.1A). Genome-wide genotyping was performed on each sample using the OmniExpress DNA Array chip, which contains approximately 500,000 markers spread across the genome. Data was uploaded in Genome Studio and checked for quality before being exported in PLINK format for downstream use in homozygosity mapping and linkage analysis.

*Homozygosity Mapping*

Although the family did not report any consanguinity, homozygosity mapping was employed for the sake of completeness. PLINK was used to identify regions of homozygosity in each sample using the default parameters. The extent of homozygosity was quantified and tracts of homozygosity shared only by affected individuals was identified that could be further scrutinised for potentially causative homozygous variants.

*Linkage Analysis*

Linkage analysis was performed using Merlin under the hypothesis of autosomal recessive inheritance with complete penetrance for individuals carrying both mutated alleles. Areas of interest were defined as those with a LOD score of greater than 0.

*Variant Filtration*

To isolate potentially pathogenic variants, we applied a systematic filtering procedure to the data (supplementary figure). We began by selecting only those variants that were present in the exome data both affected sibling for analysis. Synonymous variants (unless in a splicing region) and variants recorded in dbSNP135 were initially removed. We then filtered out any variant present at a global minor allele frequency of ≥1% in a range of publically available databases of sequence variation (1000 Genomes, Complete Genomic 69 Database and NHLBI Exome Sequencing Project database) as well as those found in 2 or more our own in-house exomes from individuals with unrelated diseases (*n*≈200).


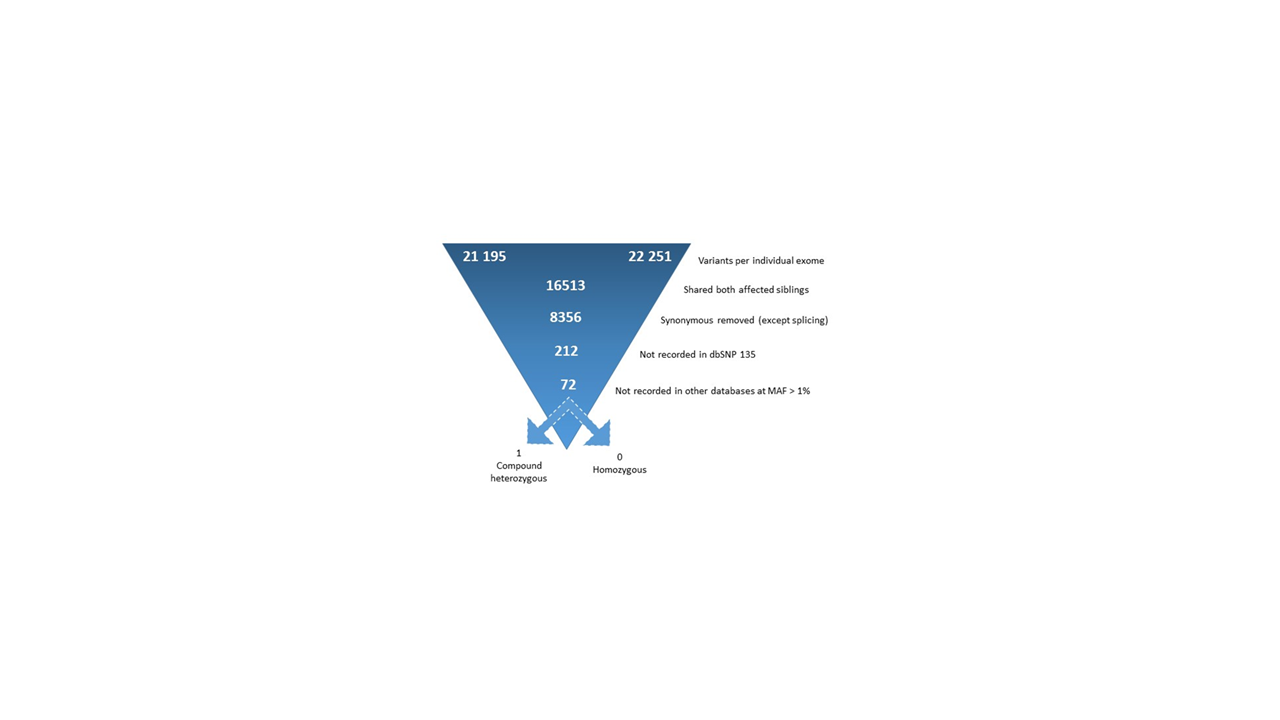


Supplementary Figure: Schematic representation of the major steps of the filtration process.

It depicts the number of variants remaining at each stage. Databases of human sequence variation used in filtration were dbSNP135, 1000 genomes, the NHLBI exome sequencing project and complete genomics 69.

**Supplementary results:**

*Exome Sequencing Coverage*

Exome sequencing produced good coverage in all three individuals. Using the CCDS hg19 definition of the exome, the average mean read depth across the entire exome was 55 with 93.5% covered by at greater than 2 reads, 81.3% covered at greater than 10 reads and 80.4% covered at greater than 20 reads. This translated to an average variant count of 22,250 variants per exome.

*Homozygosity Mapping*

Autozygosity mapping demonstrated that runs of homozygosity greater than 1Mb were both rare and small (maximum size < 3Mb), suggesting that recent consanguinity was not a feature within this family. Only three small runs of homozygosity that were shared by the two affected siblings but not present in the unaffected siblings were identified (summarized in supplementary table 1).

Within these regions, *KCND2* (a potassium channel that contributes to repolarization in cardiac cells) is the only known gene annotated on Ensembl. Manual examination of the exome data for this gene showed that it had been well covered and no potentially-causal detected. In summary, this analysis suggested that the causal variants were more likely to be compound heterozygous changes.

*Linkage Analysis*

Linkage analysis was not expected to produce a truly significant LOD score but was instead used to highlight areas with a greater likelihood of harboring a causal variant. All peaks of positive linkage shared a common maximal LOD score of approximately 0.8 and were thus all treated as areas of potential interest. The position and size of these regions is shown in supplementary table 2. Of note, there is no linkage peak over the chromosomal positions of *NUP62* (chr19, 50,410,082 - 50,432,988) or *SLC25A19* (chr17, 73,269,061 – 73,285,591), making it distinctly unlikely that these genes were responsible for disease in this family. *NDUFV1* is, however, covered by a linkage peak on chromosome 11.

*Exclusion of Genes Known to Cause Bilateral Striatal Necrosis*

No other potentially causative compound heterozygous or homozygous mutations were identified in the exome data despite good coverage. In addition, the main mitochondrial genes associated with BSN were sanger-sequenced, and no potentially causative variants were present (see supplementary table 3).

**Supplementary Tables:**

Table S1 – Results of Homozygosity Mapping

Details of the runs of homozygosity found to be shared between both affected siblings and not present in any unaffected sibling. Only one annotated gene was identified within these small regions, *KCND2*. Biological plausibility for this gene was low and inspection of the exome data showed it had been well covered without detection of any potential causal variants.

| **Chromosome** | **Start** | **Stop** | **Size (Kb)** | **Known Genes** |
| --- | --- | --- | --- | --- |
| 3 | 95058076 | 96066172 | 1008 | None |
| 7 | 118229822 | 120273226 | 2043 | *KCND2* |
| 23 | 25798188 | 27120547 | 1322 | None |

Table S2 – Results of Linkage Analysis

Physical characteristics of linkage peaks detected in this family. Given the small size of the family, multiple peaks with a maximal LOD score of 0.8 were identified, which formed the primary areas of interest.

| **Chromosome** | **Start Position** | **Stop Position** | **Size (Mb)** |
| --- | --- | --- | --- |
| 1 | 37185336 | 91528185 | 54.34 |
| 2 | 23050710 | 31066592 | 8.02 |
|  | 62430208 | 114273105 | 51.84 |
|  | 139492011 | 170146913 | 30.65 |
| 3 | 72354511 | 111138405 | 38.78 |
|  | 191860535 | 196452238 | 4.59 |
| 5 | 39087074 | 57411570 | 18.32 |
|  | 95882162 | 101831523 | 5.95 |
| 6 | 153588258 | 167018721 | 13.43 |
| 8 | 4219026 | 13521103 | 9.30 |
| 9 | 107552924 | 108893850 | 1.34 |
| 10 | 570172 | 5593361 | 5.02 |
| 11 | 45051827 | 83282729 | 38.23 |
| 12 | 3255362 | 8068881 | 4.81 |
|  | 46361672 | 66829726 | 20.47 |
| 13 | 27409049 | 30855014 | 3.45 |
| 14 | 24872917 | 33609861 | 8.74 |
| 15 | 37840246 | 85423958 | 47.58 |
| 18 | 2761338 | 11025325 | 8.26 |
| 20 | 129635 | 10968733 | 10.84 |
|  | 60216060 | 61008537 | 0.79 |
| 22 | 36545780 | 46931838 | 10.39 |

Table S3 – Mutational Analysis of common genetic causes of BSN

Results of sequence analysis of known genes most commonly associated with bilateral striatal necrosis. SLC25A19, NUP62 and NDUFV1 were analysed via the exome data. ATP6 and ND6, which are encoded by mitochondrial DNA, were sequenced using Sanger methodology. Most variants detected were non-coding. The sole coding variant detected was a common SNP present in around 30% of the population, essentially ruling it out as a cause of disease in this family.

| **Gene** | **Position** | **Base change** | **Protein change** | **SNP number** | **MAF (dbSNP)** | **Zygosity** |
| --- | --- | --- | --- | --- | --- | --- |
| SLC25A19 | 73285461 | C>A | None | rs2291033 | 0.11 | hom |
|  | 73279624 | G>C | None | rs7213318 | 0.09 | het |
|  | 73269676 | C>T | None | rs4789164 | 0.41 | hom |
| NUP62 | 50412417 | G>A | None | rs999583 | 0.77 | hom |
|  | 50412217 | C>G | S283T | rs79747934 | 0.34 | hom |
|  | 50411742 | A>G | None | rs892028 | 0.95 | hom |
| NDUFV1 | No variants detected by exome sequencing | | | | | |
| ATP6 | No variants detected by Sanger sequencing | | | | | |
| ND6 | No variants detected by Sanger sequencing | | | | | |
